# A Treatise on the Diseases of the Ox

**Published:** 1881-10

**Authors:** 


### REVIEWS.

---

A TREATISE ON THE DISEASES OF THE OX; being a Manual of Bovine Pathology; especially adapted for the use of Veterinary Practitioners and Students. By JOHN HENRY STEEL, M.R.C.S., F.Z.V.S. New York: John Wiley & Son, 13 Astor Place. 1881.

The above is the title of a most valuable contribution to bovine diseases. This branch of veterinary literature has long needed a scientific, comprehensive, and reliable treatise on the nature, pathology, surgery, and treatment of the accidents and diseases of cattle. The volume before us, of about 300 hundred pages, well and judiciously written and arranged, comes to us, therefore, in an opportune time, as the practitioners and students of this branch of comparative medicine require just what this book offers.

The introduction begins appropriately with a definition of disease and a discussion of its causes. The author then details the methods of diagnosis; and urges the necessity of obtaining a full history of the causes. In the latter chapters, medicinal agents receive full consideration. Bovine surgery is also treated of. The anatomy of the ox is briefly given.

The volume is profusely illustrated with wood-cuts, and clearly and concisely written. The arrangement of the subjects discussed is judicious; commencing with diseases of the blood, and continuing with the disease of the different systems of the body.

On the subject of contagious diseases of cattle, and the sanitary measures required for the prevention of their spread, and in the views expressed regarding the value of *inoculation* and *vaccination*, the author has shown a wide knowledge and excellent judgment.

Steel's "Diseases of the Ox" is a book indispensable to the veterinary practitioner.

